# Comparative Transcriptome and iTRAQ Proteome Analyses Reveal the Mechanisms of Diapause in *Aphidius gifuensis* Ashmead (Hymenoptera: Aphidiidae)

**DOI:** 10.3389/fphys.2018.01697

**Published:** 2018-11-30

**Authors:** Hong-Zhi Zhang, Yu-Yan Li, Tao An, Feng-Xia Huang, Meng-Qing Wang, Chen-Xi Liu, Jian-Jun Mao, Li-Sheng Zhang

**Affiliations:** Key Laboratory of Integrated Pest Management in Crops, Ministry of Agriculture, Sino-American Biological Control Laboratory, USDA-ARS/Institute of Plant Protection, Chinese Academy of Agricultural Sciences, Beijing, China

**Keywords:** *aphidius gifuensis*, diapause, molecular mechanism, transcriptome, proteome

## Abstract

*Aphidius gifuensis* Ashmead (Hymenoptera: Aphidiidae) is a solitary endoparasitoid used in the biological control of various aphids. Diapause plays an important role in the successful production and deployment of *A. gifuensis*. Diapause can effectively extend the shelf life of biological control agents and solve several practical production problems like long production cycles, short retention periods, and discontinuities between supply and demand. In recent years, studies have been conducted on the environmental regulation and physiological and biochemical mechanisms of diapause in *A. gifuensis*. Nevertheless, the molecular mechanism of diapause in this species remains unclear. In this study, we compared the transcriptomes and proteomes of diapause and non-diapause *A. gifuensis* to identify the genes and proteins associated with this process. A total of 557 transcripts and 568 proteins were differentially expressed between the two groups. Among them, (1) genes involved in trehalose synthesis such as glycogen synthase, glycogen phosphorylase, and trehalose 6-phosphate synthase were upregulated in diapause at mRNA or protein level while glycolysis and gluconeogenesis-related genes were downregulated, suggesting that *A. gifuensis* stores trehalose as an energy resource and cryoprotectant; (2) the expression of immune-related genes like C-type lectins, hemocyanin, and phenoloxidase was increased, which helps to maintain immunity during diapause; (3) a chitin synthase and several cuticular protein genes were upregulated to harden the cuticle of diapausing *A. gifuensis* larval. These findings improve our understanding of *A. gifuensis*. diapause and provide the foundation for further pertinent studies.

## Introduction

*Aphidius gifuensis* Ashmead is widely distributed in East Asian countries (Tang and Chen, 1984; Nakata, 1995). It is a common parasitoid of many aphid species. This wasp has high parasitic capacity and adaptability and has been tested as a biological control agent against the aphids *Myzus persicae* (Sulzer), *Sitobion avenae* (Fabricius), and *Aphis gossypii* (Glover) (Pan and Liu, 2014; Khan et al., 2016). It is thought that the release of *A. gifuensis* in greenhouses or open fields might control aphid populations and prevent catastrophic crop damage. In practice, *A. gifuensis* has already been used to control *M. persicae* and has proven to be highly effective (Yang et al., 2009).

Diapause is a form of dormancy that occurs before environmental conditions become unfavorable for development. It ends when favorable environmental conditions return (Denlinger and Armbruster, 2014). The diapause process consists of six phases: induction, preparation, initiation, maintenance, termination, and post-diapause development (Kostál, 2006). When insects enter diapause, their metabolic rates are depressed and their nutrient reserves are increased. In this way, they can survive predictable adverse conditions and synchronize their life cycles with the seasons conducive to growth, development, and reproduction (Hahn and Denlinger, 2011). In general, insect diapause is induced by the low temperatures and short photoperiods of late autumn in preparation for overwintering. However, several insect species enter diapause in summertime to avoid heat and drought (Saulich and Musolin, 2017). Other insects can enter both summer and winter diapause, which results in differential voltinism (Takeda, 1998; Xue et al., 2002; Ding et al., 2003; Ren et al., 2018). Diapause is of great importance in pest management because it enables the prediction of pest emergence, facilitates pest control planning, and increases the shelf life and utility of biological control agents (Denlinger, 2008). In recent years, insect diapause research has elucidated its environmental regulation (Saunders, 2014), maternal effect (Voinovich et al., 2016), energetics (Hahn and Denlinger, 2011), hormonal control (Denlinger et al., 2012), and molecular mechanisms (Hand et al., 2016).

**Figure 1:**
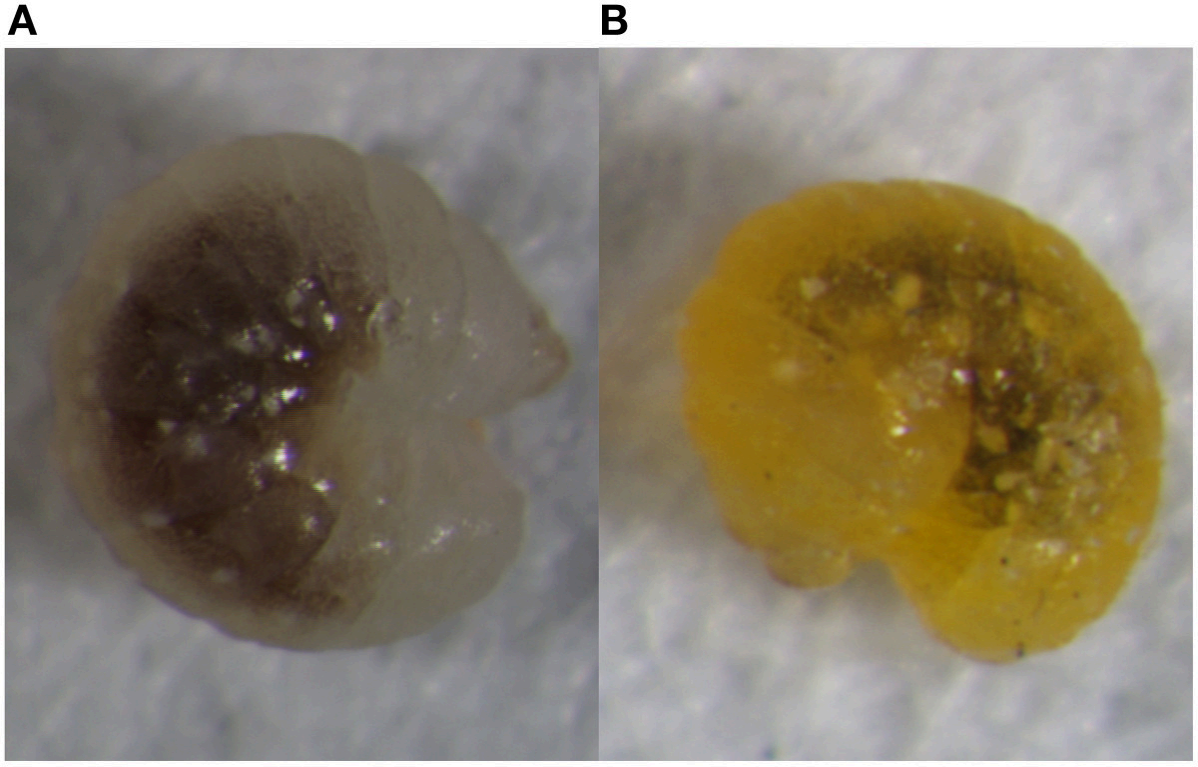
Developing **(A)** and diapausing **(B)** fourth-instar larvae of *A. gifuensis*. Of note is the substantial body color difference between these two states. The raw files of these pictures are shown as Figures S1, S2.

It is believed that the parasitoid wasp *A. gifuensis* does not enter diapause and can even develop in winter (Ohta and Ohtaishi, 2006). In China, however, several scientists found that *A. gifuensis* may enter diapause as mature larvae at low temperatures and short day lengths (Li et al., 1999; Xu et al., 2016). Our laboratory successfully induced diapause in *A. gifuensis* at 8°Cand an 8 h light:16 h dark (L8:D16) photoperiod. We found that temperature played a more vital role than photoperiod in diapause induction (Li et al., 2013). When they enter diapause, the fourth instar larvae turn from white to mustard yellow. This body color change is an important identifying characteristic of diapause in *A. gifuensis* (Figure 1). Diapausing *A. gifuensis* have higher total sugar and lower protein levels than non-diapausing individuals. Therefore, *A. gifuensis* can accumulate nutrients and protective materials when they enter diapause (Li, 2011 ). Nevertheless, the molecular processes occurring in *A. gifuensis* diapause have not yet been determined.

In this study, we used high-throughput mRNA sequencing (RNA-seq) and isobaric tags for relative and absolute quantification (iTRAQ) technology to analyze the transcriptomes and proteomes, respectively, in *A. gifuensis* diapause (D) and non-diapause (ND) fourth instar larvae. We compared their relative gene and protein expression levels. We determined the functions of their differentially expressed genes (DEGs) and proteins (DEPs) using the gene ontology (GO) and Kyoto Encyclopedia of Genes and Genomes (KEGG) databases. We then compared transcriptome and proteome data between the D and ND groups and selected the DEGs with either the same or inverse change trends. We also ran quantitative real-time PCR (qRT-PCR) to verify the accuracy of the transcriptome analyses.

## Methods

### Insect rearing and treatment

The green peach aphid *M. persicae* was reared on tobacco (*Nicotiana tabacum*) seedlings and used as a host for the aphid parasitoid wasp *A. gifuensis*. The tobacco, aphids, and parasitoid wasps were obtained from Langfang Pilot Scale Base, Institute of Plant Protection, Chinese Academy of Agricultural Sciences, Hebei Province, China. The aphids were reared in homemade cages at 25°C, 80% RH, and L16:D8. Adult *A. gifuensis* were released at a ratio of one parasitoid to 100 aphids. Daily observations were made and recorded. Mummies were dissected and fourth instar larvae were used as ND samples. Certain aphids were transferred to diapause-inducing conditions (8°C, RH 80%, and L8:D16) 3 d after being parasitized. Once again, mummies were dissected and fourth instar larvae were used as D samples.

### RNA preparation, cDNA library construction, and illumina sequencing

Three replications were performed under each treatment and 50 larvae were used in each replication. Total RNA was extracted using RNAiso Plus Total RNA extraction reagent (TaKaRa Bio Inc., Kusatsu, Shiga, Japan) following the manufacturer's instructions. An Agilent Bioanalyzer 2100 (Agilent Technologies, Santa Clara, CA, USA) and the RIN number were used to evaluate RNA integrity. Qualified total RNA was further purified with the RNeasy micro kit (Qiagen, Hilden, Germany) and the RNase-Free DNase Set (Qiagen, Hilden, Germany). Total RNA was extracted and oligo (dT) magnetic beads and fragmentation buffer were used to generate short fragments of the isolated poly(A) mRNA. First-strand cDNA was generated using SuperScript II Reverse Transcriptase (Invitrogen, Carlsbad, CA, USA) with these short fragments and random hexamer primers. Second-strand cDNA synthesis was performed using the Second Strand cDNA Synthesis Kit (Beyotime, Shanghai, China). The adapter was ligated immediately after repair of the adenylated 3′ ends and purification with a QIAquick PCR purification kit (Qiagen, Hilden, Germany). A cDNA template enrichment was required for library QC. Concentration and library size were assessed using a Qubit 2.0 fluorometer (Invitrogen, Carlsbad, CA, USA) and an Agilent 2100 Bioanalyzer (Agilent Technologies, Santa Clara, CA, USA), respectively. Three biological replicates of the non-diapause and diapause samples were sequenced with an Illumina HiSeq 2500 platform (Illumina, San Diego, CA, USA). Paired-end sequencing was controlled with the data collection software provided by Illumina using real-time data analysis.

### Transcript identification and quantification

The cDNA sequencing was performed using the Illumina HiSeq 2500 Sequencing System (Illumina, San Diego, CA, USA) according to the manufacturer's instructions. The clean reads were filtered from the raw reads using fastx v. 0.0.13 (http://hannonlab.cshl.edu/fastx_toolkit/index.html) to eliminate ribosomal RNA reads, adapter sequences, reads with more than 20% low quality bases, reads shorter than 50 bp, and reads with an N (unknown sequences) ratio>5% (Patel and Jain, 2012). *De novo* clean read assembly was performed using the scaffolding contig algorithm of CLC Genomics Workbench v. 6.0.4 according to the sequencing data of six samples (Bräutigam et al., 2011; Garg et al., 2011; Su et al., 2011). The primary unigenes obtained from the *de novo* assembly were then expressed sequence tag (EST)-assembled by CAP3. The final unigenes were then searched against the UniProt and Nr database with BLASTx. The best hits from the database were selected as unigene annotations. For quantitative gene expression analysis, reads from each sample were mapped to the final unigenes which acted as reference sequences. The read counts were normalized by trimmed mean M values (TMM; Robinson and Oshlack, 2010), and then a standard calculation was performed based on reads per kilobase of transcript per million mapped reads (RPKM; Mortazavi et al., 2008). The expressions of transcripts are summarized in Table S1.

### Protein extraction

Three replicates (50 larvae for each replication) were used for the D and ND treatments. Samples were quickly ground into a very fine powder in liquid nitrogen and added to centrifuge tubes containing 1 ml ice-cold BPP buffer [100 mm Tris, 100 mm ethylenediaminetetraacetic acid (EDTA), 50 mm borax, 50 mm vitamin C, 1% PVPP w/v, 1% Triton X-100 v/v, 2% β-mercaptoethanol v/v and 30% sucrose w/v, pH 8.0]. After the suspension was vortexed at room temperature for 10 min, 800 μl Trissaturated Phe were added and further vortexed for 10 min. The homogenates were then centrifuged at 15,000 × *g* and 4°C for 15 min. The supernatants were then transferred to new centrifuge tubes (200 μl supernatants per tube). 1ml ammonium sulfate saturated-methanol was added to each tube and then the mixture was incubated over night at −20°C. After being spun at 15,000 × *g* and 4°C for 15 min, the precipitates were then re-suspended in 500 μl ice-cold methanol, and then centrifuged at 15,000 × *g* and 4°C for 5 min. 500 μl ice-cold methanol acetone was added to the precipitates and centrifuged as described above. The protein precipitates were air-dried at room temperature and dissolved in 200 μl lysis buffer [50 mm Tris, 1 mm phenylmethanesulfonyl fluoride (PMSF), 2 mm ethylenediaminetetraacetic acid (EDTA), and 2 mm dithiothreitol (DTT), pH 7.4] at 22°C for more than 2 h. The homogenates were then centrifuged at 17,000 × *g* and 20°C for 30 min. The protein samples (supernatant) were packed into 1.5-ml microcentrifuge tubes and stored at −80°C. Protein concentrations were determined by the Bradford method (Bradford, 1976). Each treatment included three biological and three technical repetitions.

### ITRAQ LABELING and UPLC-QTOF-MS/MS analysis

200 μg protein from each sample solution was transferred to the centrifuge tube and diluted to 125 μl with 8 M UA buffer (8 M urea and 150 mm Tris-HCl, pH 8.0). 5 μl 1M DTT was added to the homogenates which were then incubated at 37°C. After 1 h, 20 μl1 M iodoacetamide (IAA) were added to each centrifuge tube. These were incubated for 1 h in a dark room. The samples were then transferred to an ultrafiltration centrifuge tube and spun at 14,000 × *g* and room temperature for 20 min. The supernatants were then discarded. 100 μl 1M UA buffer was added to an ultrafiltration centrifuge tube and spun at 14,000 × *g* room temperature for 10 min. This step was repeated twice. One hundred microliters of dissolution buffer (AB SCIEX, Framingham, MA, USA) was added to an ultrafiltration centrifuge tube and spun at 14,000 × *g* room temperature for 20 min. This step was repeated three times. Samples were then transferred to new collection tubes. Protein samples were digested at 37°C overnight with sequencing-grade trypsin (Worthington Biochemical, Lakewood, NJ, USA) at a protein:trypsin ratio of 50:1. After centrifugation at 14,000 × *g* room temperature for 20 min, the peptides were collected and the remaining homogenates were recentrifuged with 50 μl dissolution buffer. The filtrates were pooled with those from the previous step. The iTRAQ labeling was performed with an iTRAQ Reagent-8plex Multiplex Kit (AB SCIEX, Framingham, MA, USA) according to the manufacturer's protocol. Six samples (three replicates each for ND and D) were labeled with iTRAQ reagent as follows: 113 (ND1), 114 (ND2), 115 (ND3), 116 (D1), 117 (D2), and 118 (D3). After labeling, the peptides were fractionated by reversed phase liquid chromatography (RPLC) on a Gemini-NX C18 (4.6 mm × 150 mm, 5 μm; Phenomenex, Aschaffenburg, Germany) to remove any remaining iTRAQ or other reagents. MS was performed using an AB SCIEX TripleTOF^TM^ 5600(AB SCIEX, Framingham, MA, USA) coupled online with the LC-20AD Nano-HPLC system (Shimadzu Corp., Kyoto, Japan). The gas setting of the MS was as follows: curtain gas = 35 kPa; Gas1 = 4; Gas2 = 0. The ion spray floating voltage was 2,300 V and the collision energy voltage used for collision-induced dissociation (CID) fragmentation for MS/MS spectra acquisitions was 80 V. Each cycle consisted of a TOF-MS spectrum acquisition for 250 ms. The mass-to-charge ratio (m/z) scan range was 350-1,250 Da. There were 30 information-dependent acquisitions (IDA; m/z = 100-1,500 Da). The accumulation time of each IDA was 0.1 s. Masses were dynamically excluded for 25 s when MS/MS fragment spectra were acquired for them. MS was recalibrated at the start of each sample with a β-galactosidase digest standard.

### Protein identification and quantification

Protein identification and quantification were performed with ProteinPilot 4.5 (AB SCIEX, Framingham, MA, USA). Raw MS/MS data were searched against the UniProt database. Peptides with unused scores ≥1.3 (corresponding to a confidence limit of 95%) were accepted. Predicted proteins with ≥1 unique peptide and a global false discovery rate (FDR) ≤ 1% were counted as identified. The quantitative result was outputted as ratios of proteins in every labeled sample to it in sample 113. A statistical comparison of the differences in protein expression between diapaused and non-diapaused *A. gifuensis* was conducted with one-way ANOVA in SPSS v. 13.0 (IBM Corp., Armonk, NY, USA). *P*-values were calibrated by the Benjamini method (Benjamini and Hochberg, 1995).

### Bioinformatics analysis

The identified transcripts and proteins were classified according to the Gene Ontology (GO; Ashburner et al., 2000) database using BLAST (E-value < 1e-5). KAAS (http://www.genome.jp/tools/kaas/) was used to map gene and protein pathways based on the Kyoto Encyclopedia of Genes and Genomes (KEGG; Kanehisa and Goto, 2000) database. Enrichment analyses were performed on the differentially expressed genes (DEGs) and differentially expressed proteins (DEPs) based on the GO and KEGG annotations. DAVID v. 6.7 (https://david-d.ncifcrf.gov/) was used for these analyses.

### Quantitative real-time PCR

The qRT-PCR was carried out in a 7,900 HT Sequence Detection System (Applied Biosystems, Foster City, CA, USA) using the ABI Power SYBR Green PCR Master Mix (Applied Biosystems, Foster City, CA, USA). Glyceraldehyde-3-phosphate dehydrogenase (GAPDH) was used as a reference gene for relative target gene quantification. The target genes included the upregulated fatty acid synthase (FAS), gamma-interferon-inducible-lysosomal thiol reductase (GILT), cuticle protein 21 (CP21), uncharacterized protein LOC103571003, serine protease homolog 21 precursor (SP21), trehalose phosphate synthase (TPS), troponin C (TnC), sodium/potassium-transporting ATPase (AT1A), and the downregulated venom carboxylesterase-6 (EST6). The primer sequences of GAPDH and the nine target genes are listed in Table 1. Nine replicates (three biological replicates × three technical replicates) were used per sample. The qRT-PCR conditions were as follows: incubation at 50°C for 2 min and 95°C for 10 min followed by 40 cycles of 95°C for 15 s and 60°C for 1 min. To detect non-specific product amplification, the melting curve was analyzed from 60°C to 95°C. The amplification efficiency was automatically calculated using SDS v. 2.4 (Applied Biosystems, Foster City, CA, USA). Relative quantification of the target gene transcripts was determined by the comparative CT method 2^−Δ*ΔCt*^ (Livak and Schmittgen, 2001).

**Table 1:** Primers used for qRT-PCR.

**Gene name**	**Primer name**	**Primer sequence**
*Fas*	FAS-F	TTCCCACAGGCTATCTTTCA
	FAS-R	TGGCAAAGAAGCTTACAATCG
*GILF*	GILF-F	TGAGCTTTATTTCCAGCACATTCT
	GILF-R	ACGCACAAGTATGATGAATCAACTG
*EST6*	EST6-F	GGTCTTTACCCAGGTGCTGA
	EST6-R	AAATGGTGCAAGTTCGTCGT
*CP21*	CP21-F	TGGCTAATTCTTCAGCAGCA
	CP21-R	CGTTCATCTTCAGGCATTGTT
*LOC103571003*	LOC-F	TCCACGTTGTCCAGTTTGTC
	LOC-R	ATGACGTTGTACTTCATCAGCAAC
*SPH21*	SPH21-F	GGAAATTCACCAAATTGTGC
	SPH21-R	GACCACCACCATTTCAATCAC
*TPS*	TPS-F	CGTTCATCTTCAGGCATTGTT
	TPS-R	GCTGGAGCTGGTGAAATGAT
*TnC*	TnC-F	ATTGAACACGAGTACTGAATGATTGAT
	Tnc-R	CGCAACAGCTATACCATGAGCTT
*AT1A*	AT1A-F	TGTATTTCTTTGGCAATTGGTGTT
	AT1A-R	TGGTGATCAAACTGTAATGGGTAGA
*GAPDH*	GAPDH-F	AAAGTTAAGGAAGCTGCTGCAC
	GAPDH-R	GGCATCAAAAATACTTGAGTGAGT

## Results

### Summary of transcriptome data

The RNA-seq experiment was performed on both non-diapaused and diapaused *A. gifuensis* according to the data obtained by sequencing with the Illumina HiSeq 2,500 platform. Using three biological replicates of two samples, the six libraries yielded 230,886,306 raw reads comprising 154,790,202 for the ND group and 145,249,536 for the D group. After quality filtering and fragment assembly, 47,077 unigenes were obtained. The assembled unigenes were mostly 200-1,000 bp long (70.49%). Another 17.47% were 1,001-2,000 bp long and 12.05% of them were >2,000 bp. Transcriptome data were deposited in the Sequence Read Archive (SRA, https://www.ncbi.nlm.nih.gov/sra) of the National Center for Biotechnology Information (NCBI) under the accession numbers SRR3717246 (ND) and SRR3717412 (D). Transcriptome datasets are also available at NCBI project PRJNA326701 under the accession number SRP077084.

**Figure 2:**
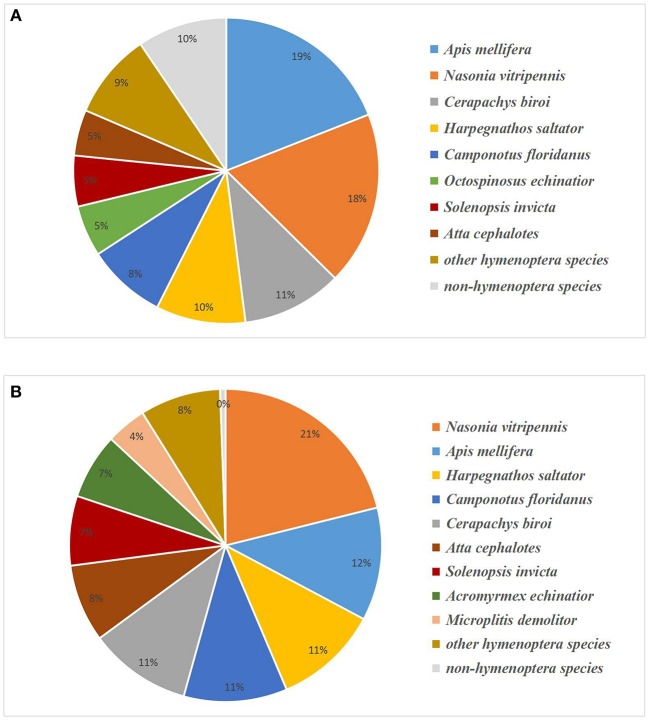
Species distribution of BLASTX hits of experimental unigenes **(A)** and proteins **(B)**. Unigenes or proteins homologous to Hymenoptera were assumed to belong to *A. gifuensis*.

We annotated the unigenes by aligning their sequences against the UniProt database with BLASTX (Altschul et al., 1990). There were 24,431 unigenes matched with a high homology (*E*-value < 1e-5; Table S2). Since the genome sequence of *A. gifuensis* was not yet determined, we performed a sequence alignment with known genomes of other species to establish whether the unigene belonged to *A. gifuensis*. BLAST results showed the greatest number of hits for *Apis mellifera* (19.03%) and *Nasonia vitripennis* (18.36%), followed by *Cerapachys biroi* (10.60%), *Harpegnathos saltator* (9.47%), *Camponotus floridanus* (8.37%), and *Acromyrmex octospinosus* (5.39%). Another 9.48% of the unigenes were found for species not belonging to the Hymenoptera (Figure 2A). These were treated as contaminants and filtered out.

In the differential expression analysis, transcripts with ≥ 1.25-fold change between ND and D and FDR (false discovery rate) ≤ 0.05 were considered DEGs. The expression levels of 557 transcripts were significantly changed; 389 were upregulated and 168 were downregulated.

### Protein identification and quantification

We identified 4,931 proteins from a total of 30,332 unique peptides using ProteinPilot v. 4.5. All of them had ≥1 unique peptide and 80.17% of them had ≥2 unique peptides. Of these, 2,852 proteins were quantified with iTRAQ ratios (Table S3). Species annotation showed that most of the matches belonged to *Nasonia vitripennis* (21.11%), *Apis mellifera* (11.68%), *Harpegnathos saltator* (10.83%), *Camponotus floridanus* (10.69%), and *Cerapachys biroi* (10.59%). Another 0.56% of the proteins were filtered out as contaminants (Figure 2B). The proteome datasets are available at Peptide Atlas (http://www.peptideatlas.org) under submission number PASS00902.

**Figure 3:**
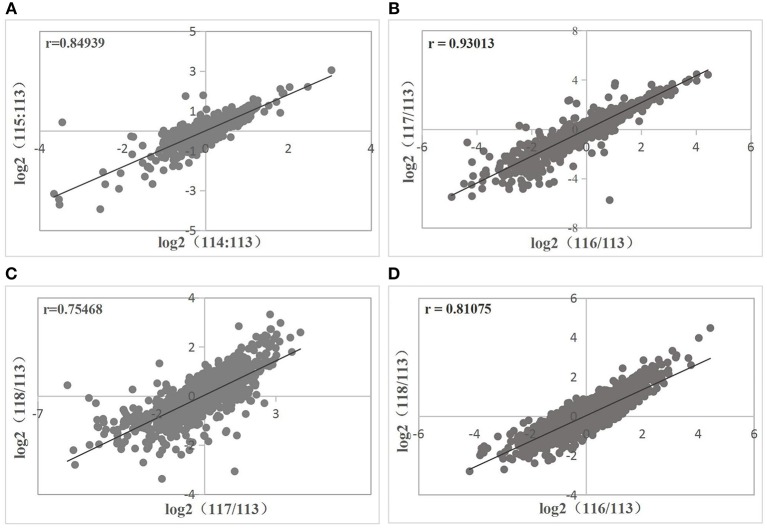
Correlations between biological replicates in ND group **(A)** and D group **(B–D)**. Correlations are described by the Pearson correlation coefficient r, where *r* > 0.6 means a strong correlation between two sets of variates.

Correlations between biological replicates were evaluated before the differential expression analysis. Sample 113, which acted as the reference sample in protein quantification, was used as the denominator. The ratios from same treatment (ND or D) were log-transformed and plotted against each other. All correlations between the two biological replicates were >0.6 (Figure 3). Therefore, reproducibility among the three replicates per treatment was satisfactory. Proteins with the same trend in all six comparisons and with average ≥1.25-fold change and FDR ≤ 0.05 were deemed DEPs. There were 253 upregulated and 315 downregulated DEPs.

### Correlation analysis of transcriptome and proteome data

**Figure 4:**
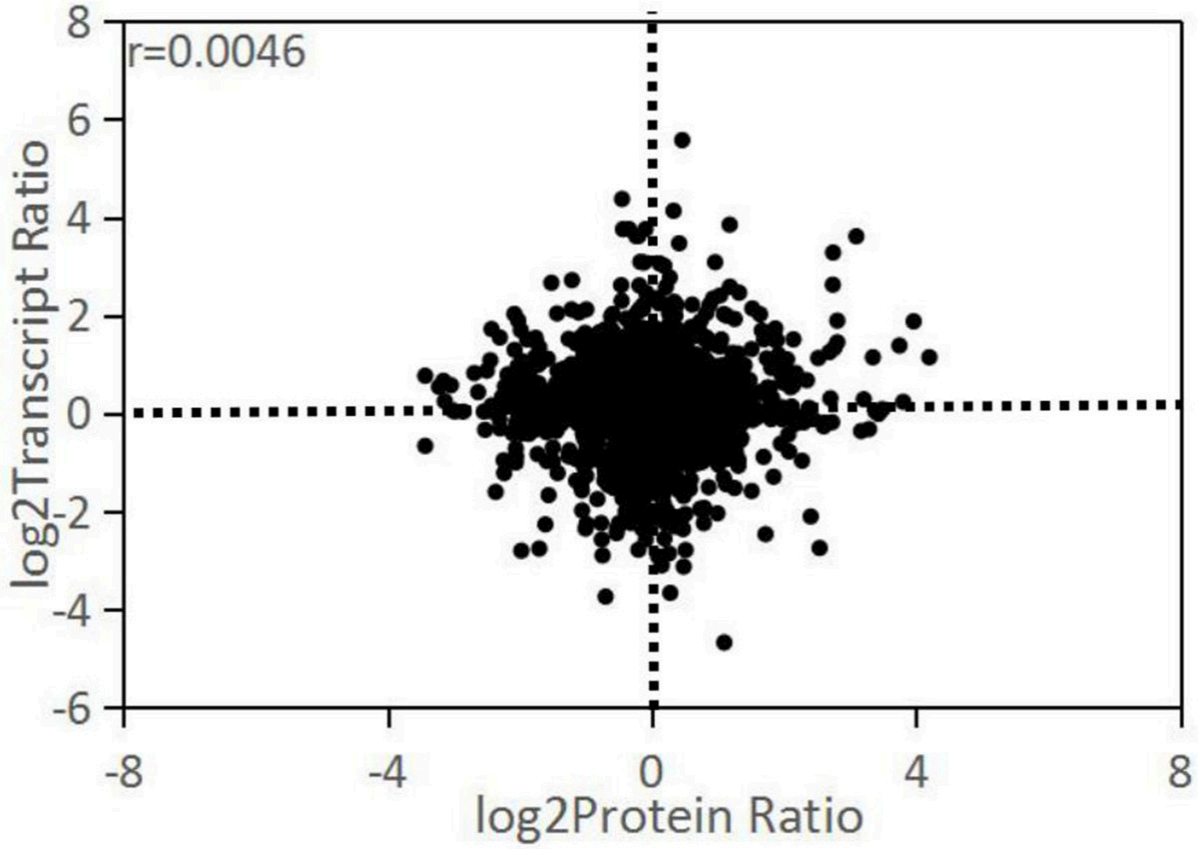
Comparison of differences in transcript and cognate protein expression levels.

There were 2,527 out of 2,852 proteins matching the corresponding genes by sequence alignment. The expression change ratios (D/ND) between transcriptome and proteome level were compared with correlation analysis. The result showed a low Pearson correlation coefficient between these two levels (*r* = 0.0046; Figure 4). All DEPs with ≥1.25-fold change matched their corresponding genes, while most of them did not significantly change. As shown in Table 2, 28 of the DEPs had the same change trend as their corresponding DEGs. Of these, 25 were upregulated and 3 were downregulated. Twelve DEPs had change trends that were the inverse of those for their corresponding DEGs.

**Table 2:** Correlated protein and mRNA pair expression comparisons.

**Protein ID**	**Gene ID**	**Species**	**log2(fold change)**	
			**Protein**	**Transcript**
H9K416_APIME	gi|340719754	*Apis mellifera*	2.14	1.93
E2AX16_CAMFO	gi|665804818	*Camponotus floridanus*	2.73	1.33
E2ADG5_CAMFO	gi|665815415	*Camponotus floridanus*	1.20	1.96
A0A026WTR5_CERBI	gi|91076412	*Camponotus floridanus*	1.30	1.13
E2B637_HARSA	gi|665796638	*Harpegnathos saltator*	1.26	2.02
E2BN65_HARSA	gi|665804353	*Harpegnathos saltator*	1.33	2.70
E2BY77_HARSA	gi|572316213	*Harpegnathos saltator*	3.49	3.09
A9YME5_MICHY	gi|380022226	*Microplitis demolitor*	3.75	1.38
A0A022T5Y4_9HYME	gi|340709090	*Microplitis demolitor*	3.91	1.78
A0A022T365_9HYME	gi|665790675	*Microplitis demolitor*	2.60	1.14
A0A022T6M5_9HYME	gi|665785537	*Microplitis demolitor*	3.10	3.61
K7J8I6_NASVI	gi|665796266	*Nasonia vitripennis*	1.36	1.43
K7ITP5_NASVI	gi|645000995	*Nasonia vitripennis*	2.54	1.45
K7JCI5_NASVI	gi|665808850	*Nasonia vitripennis*	2.69	1.25
K7IWS2_NASVI	gi|665786561	*Nasonia vitripennis*	1.09	3.09
K7J590_NASVI	gi|48097532	*Nasonia vitripennis*	1.92	1.20
K7IRC8_NASVI	gi|572267032	*Nasonia vitripennis*	2.75	3.28
K7IM71_NASVI	gi|665815415	*Nasonia vitripennis*	1.28	1.96
K7IMV0_NASVI	gi|665806184	*Nasonia vitripennis*	2.22	2.22
K7J3Q5_NASVI	gi|340723203	*Nasonia vitripennis*	2.14	1.51
K7J9P4_NASVI	gi|239049675	*Nasonia vitripennis*	1.63	1.66
K7J3V6_NASVI	gi|665810610	*Nasonia vitripennis*	1.74	1.12
E9IN39_SOLIN	gi|665796076	*Solenopsis invicta*	2.38	1.51
E9INZ6_SOLIN	gi|665807963	*Solenopsis invicta*	3.97	1.27
E9IF52_SOLIN	gi|383849856	*Solenopsis invicta*	1.49	10.11
E2AKQ3_CAMFO	gi|665813243	*Camponotus floridanus*	−2.24	−1.21
K7IPY1_NASVI	gi|665803681	*Nasonia vitripennis*	−1.03	−3.24
Q5I206_LYSTE	gi|641652645	*Lysiphlebus testaceipes*	−1.98	−2.80
H9KBS5_APIME	gi|380016708	*Apis mellifera*	−1.15	5.53
I7JI13_APIME	gi|345480545	*Apis mellifera*	−1.43	1.77
W4VY26_ATTCE	gi|350402038	*Atta cephalotes*	−1.90	5.85
E2ANM7_CAMFO	gi|345480545	*Camponotus floridanus*	−2.43	1.77
E2AHX2_CAMFO	gi|665819142	*Camponotus floridanus*	−1.50	6.57
A0A026WCA1_CERBI	gi|229608897	*Cerapachys biroi*	−2.08	2.17
A0A026W0A3_CERBI	gi|345481521	*Cerapachys biroi*	1.51	−3.66
A0A026W577_CERBI	gi|665806090	*Cerapachys biroi*	−1.08	2.12
K7IRT4_NASVI	gi|665815631	*Nasonia vitripennis*	−2.30	1.55
K7J4F5_NASVI	gi|383859846	*Nasonia vitripennis*	−3.43	1.01
K7J4F3_NASVI	gi|665792884	*Nasonia vitripennis*	−1.12	6.22
E9J2G3_SOLIN	gi|383856273	*Solenopsis invicta*	−1.08	1.56

### Functional analysis of differentially expressed transcripts and proteins

To explore the biochemical reactions involved in *A. gifuensis* diapause, an enrichment analysis based on GO annotations was performed using hypergeometric testing to categorize the DEPs and DEGs. There were 168 (62 biological processes, 94 molecular functions, and 12 cellular components) and 167 (53 biological processes, 86 molecular functions, and 28 cellular components) enriched GO terms. These classified 152 DEGs and 181 DEPs, respectively. One gene or protein could have >1 GO annotation (Tables S4, S5). The same enrichment analysis was performed based on the KEGG annotation. At the mRNA level, 80 differentially expressed genes mapped to 143 pathways (Table S6). The greatest number of DEGs were linked to “metabolic pathways,” (20) followed by “biosynthesis of secondary metabolites” (12) and “biosynthesis of antibiotics” (11). At the protein level, eight differentially expressed proteins (DEPs) mapped to 37 pathways (Table S7). The pathways with the most DEPs were “metabolic pathways,” (7) “biosynthesis of secondary metabolites,” (6) and “glycolysis/gluconeogenesis” (4). The aforementioned results suggest that glycometabolism, immunoreaction, and secondary metabolite accumulation play important roles in diapause.

### Validation of differentially expressed genes using QRT-PCR

**Figure 5:**
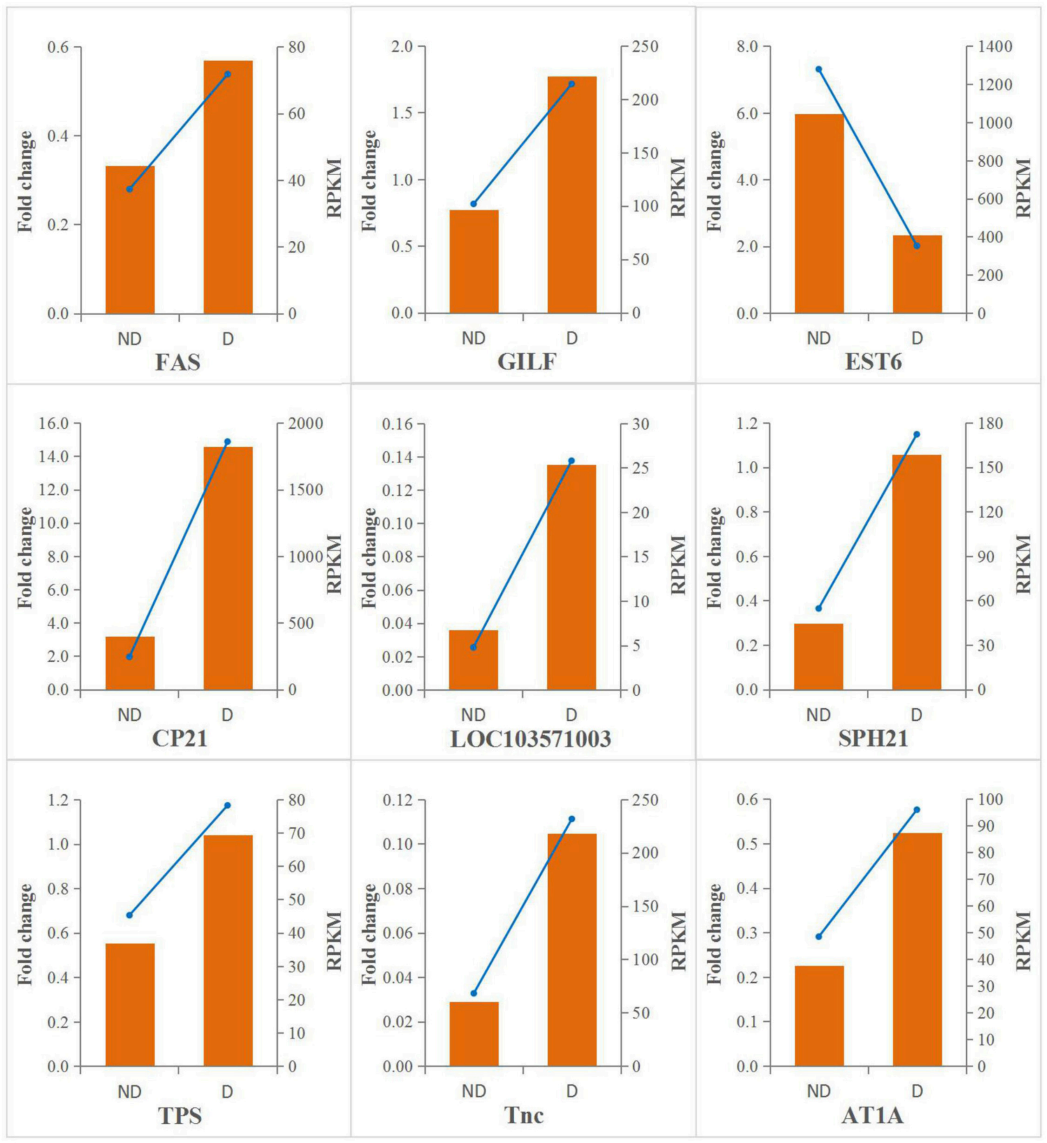
qRT-PCR validation of differentially expressed genes. The qRT-PCR results are shown in orange, and the values are shown on the left y-axis; the R result are shown with blue lines, and the value correspond to the right y-axis.

A qRT-PCR analysis was performed to investigate the transcriptional patterns of nine selected genes. As shown in Figure 5, the expression levels of all target genes were consistent between the qRT-PCR and RNA-seq data. This showed that the quality of transcriptome data was reliable.

## Discussion

### Metabolite accumulation in diapause

During diapause, an insect may feed little or not at all. For this reason, insects must store nutrients in preparation for diapause (Hahn and Denlinger, 2007). Certain metabolites like polyhydric alcohols and sugars can also serve as cryoprotectants that improve cold endurance in diapausing insects (Block, 1990). The insulin-signaling pathway may be a major player in diapause metabolite regulation (Hahn and Denlinger, 2011). The transcriptome analysis showed that the expression of the 3-phosphoinositide-dependent protein kinase-1 (PDK1) gene (*PDK1*, contig_31661) was upregulated 4.08-fold in the D group. PDK1 is an indispensable component of the PI3K-AKT signaling axis which, in turn, is a part of the insulin-signaling pathway. PDK1 phosphorylates and activates serine/threonine-protein kinase (AKT), which regulates glucose homeostasis (Sarbassov et al., 2005). The forkhead of the transcription factor Foxo1 is downstream of AKT and may regulate glucose homeostasis in insects (Sim and Denlinger, 2013). In the nucleus, FOXO1 binds the promoter of the gene encoding phosphoenolpyruvate carboxykinase (PEPCK) and activates the expression of this enzyme (Puigserver et al., 2003). Activated AKT phosphorylates FOXO1. Thence, FOXO1 is exported from the nucleus and retained in the cytoplasm, and target gene expression is repressed (Biggs et al., 1999). PEPCK converts oxaloacetate to phosphoenolpyruvate. This reaction is generally considered to be the first committed step in gluconeogenesis (Rognstad, 1979). At the mRNA level, the expression of *PEPCK* (contig_30482) was halved in the D group. Therefore, gluconeogenesis was probably inhibited in them. Another important gene downstream from AKT is glycogen synthase kinase 3 beta (GSK3β), which is involved in glycogen biosynthesis and metabolism. GSK3β inhibits glycogen synthase (GYS) by phosphorylating it. In this way, it inhibits glycogen biosynthesis (Cohen and Frame, 2001). By inhibiting GSK3β, AKT can overcome this repression and increase GYS activity (Beaulieu et al., 2009). In this study, GSK3β (K7JBD0_NASVI) was downregulated at the protein level and *GYS* (contig_31002) was upregulated at the mRNA level in the D group. Following this, glycogen synthesis was increased during *A. gifuensis* diapause.

It is generally accepted that glycogen is converted into polyhydric alcohols or trehalose in diapausing insects (Hayakawa and Chino, 1982). This transformation starts with phosphorolysis catalyzed by glycogen phosphorylase (GP). The product is *D*-glucose-1-phosphate (Steel, 1982). There are two alternative metabolic pathways for *D*-glucose-1-phosphate. In one scenario, it is converted into *D*-glucose-6-phosphate via phosphoglucomutase (PGM). In turn, *D*-glucose-6-phosphate may be transformed via glycolysis into pyruvate which would enter the TCA cycle. Glycerol and sorbitol may also be produced by glycolysis in diapausing insects (Chion, 1958). Contrarily, *D*-glucose-1-phosphate could be converted into trehalose via UTP-glucose-1-phosphate uridylyltransferase (UGP), trehalose 6-phosphate synthase (TPS), and trehalose 6-phosphate phosphatase (TPP). Several studies have shown that independent *TPP* genes were absent in insects whereas there were *two* conserved regions corresponding to *TPS* and *TPP*, respectively (Cui and Xia, 2009; Xu et al., 2009; Tang et al., 2010). In the D group, the expression levels of *GP* (contig_3475) and *TPS* (contig_3499) were 1.29-fold and 1.73-fold upregulated, respectively, while the protein pgm (K7IN84_NASVI) was downregulated 0.80-fold. Therefore, we speculate that diapausing *A. gifuensis* decreases energy expenditure and stores nutrients in the form of trehalose rather than glycerol or sorbitol.

**Figure 6:**
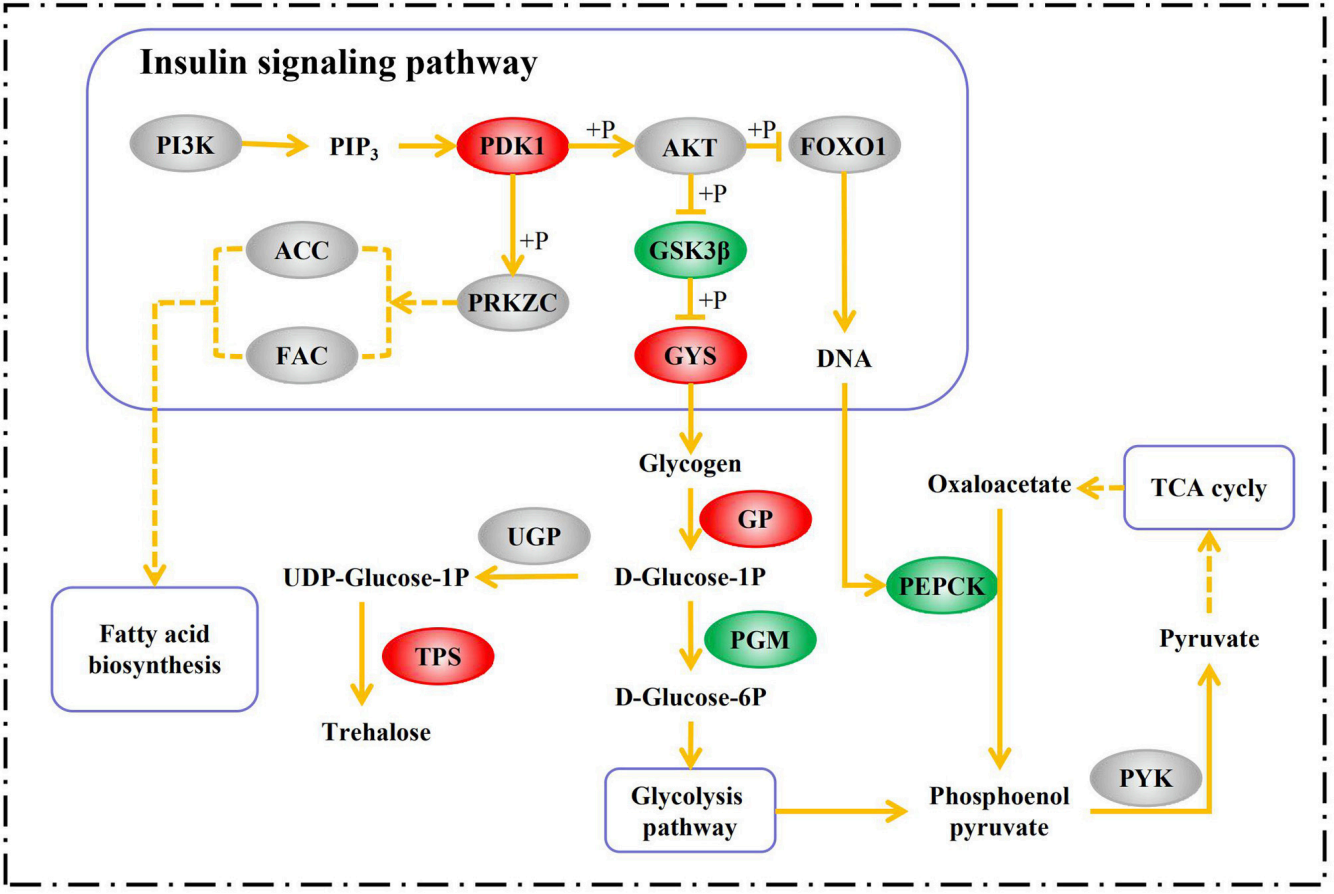
Summary of the pathways involved in metabolite accumulation in *A. gifuensis* diapause. Red boxes indicate genes/proteins upregulated in the D group relative to the ND group. Green boxes indicate genes/proteins downregulated in the D group compared with the ND group. Gray boxes indicate genes/proteins not differently expressed in the D group in comparison with the ND group. PI3K, phosphatidylinositol-4,5-bisphosphate 3-kinase; PIP3, Phosphatidylinositol-3,4,5-trisphosphate; PRKZC, atypical protein kinase C zeta type; PYK, pyruvate kinase.

Several insects like *Colaphellus bowringi* (Tan et al., 2017), *Pieris napi* (Lehmann et al., 2016), *Arimania comaroffi* (Bemani et al., 2012), and *Ectomyelois ceratoniae* (Heydari and Izadi, 2014) accumulate lipids in diapause whereas others like *Cymbalophora pudica* (Kostál et al., 1998) and *Eurytoma amygdali* (Khanmohamadi et al., 2016) do not. In group D, fatty acid synthase (FAS, K7IM26_NASVI) and acetyl-CoA carboxylase (ACC, E2B9B3_HARSA) were non-significantly downregulated at the protein level (0.88- and 0.89-fold change, respectively). The result suggested that *A. gifuensis* relies on sugars like trehalose rather than lipids as energy sources and cryoprotectants in diapause. The aforementioned signals and metabolic processes are summarized in Figure 6.

### Immune responses in diapause

Comparatively little research has been conducted on immune responses in diapausing insects. Metabolic and developmental retardation in diapause are expected to weaken insect immune systems. However, diapause must not lower insect defenses against pathogens if it is to ensure insect survival under harsh environmental conditions. A report on *Samia cynthia* suggested that immunity continues to work well-during diapause (Nakamura et al., 2011). Insects may maintain immunity during diapause by regulating the expression of certain proteins. In the D group, pattern recognition proteins, C-type lectins (CTL, A0A088ACU2_APIME), and the immune globulin hemocyanin (HC, F4X264_ACREC) were all upregulated at the protein level and the humoral immune-related protein phenoloxidase (*PO*, contig_33789) was upregulated at the mRNA level.

C-type lectins are a superfamily of proteins binding and agglutinating bacterial lipopolysaccharides and lipoteichoic acids (Dodd and Drickamer, 2001). Reports on endoparasitoid wasps indicated that CTL binds parasitoid eggs to mask their hemocyte-binding sites. In this way, the wasp could evade detection by the host immune system (Glatz et al., 2003; Lee et al., 2008; Nalini et al., 2008). Since *A. gifuensis* enter diapause as fourth instar larvae, however the host is already dead by that time. Therefore, the role of CTL upregulation in *A. gifuensis* diapause is to improve pathogen defense.

Hemocyanin is the major protein component of invertebrate hemolymph. It participates in non-specific invertebrate immunity. A study on *Scylla serrata* suggested that HC agglutinates bacteria in preparation for host cell defensive measures (Yan et al., 2011). Zhang et al. (2004) reported that purified HC had non-specific antiviral properties. In response to microbial infection, HC can also be converted by proteolytic cleavage into antimicrobial peptides (Destoumieux-Garzón et al., 2001). Since hemocyanin is multifunctional, the 8.45-fold upregulation of HC expression in diapausing *A. gifuensis* may have been required for protein storage (Jaenicke et al., 1999), ecdysis regulation (Paul et al., 1994), and other processes besides immunity enhancement.

Phenoloxidase is an important enzyme in melanization. It oxidizes phenolic molecules to produce melanin around invading pathogens and wounds (Lu et al., 2014). Melanin and the melanization intermediates may be cytotoxic to microorganisms and impede pathogen invasion. They may also participate in insect wound healing (Eleftherianos and Revenis, 2011). In the D group, there was a 2.93-fold upregulation of phenoloxidase, which was manifested through increased melanin biosynthesis. These findings suggest that insects regulate immunity in diapause by accumulating immune-related proteins. Nevertheless, the precise nature of the changes in cellular immunity that occur during diapause remains unknown.

### Melanism and cuticle sclerotization in diapause

The cuticle is the protective barrier between the internal tissues and the external environment in holometabolous larval insects. It is composed of chitin fibers and cuticle proteins (Charles, 2010). Cross-links between the fibers and proteins result in hardening, dehydration, and close packing. This process and cuticle pigmentation are collectively referred to as tanning (Arakane et al., 2005). PO is present during the formation of the cuticulin layer. The quinones generated by PO cross-link the protein chains and stabilize the cuticle (Locke and Krishnan, 1971). A study on *Bombyx mori* reported that melanization occurred in the cuticle (Ashida and Bery, 1995). Therefore, melanin can form from quinones in the cuticle. Phenoloxidase upregulation, then, may explain the body color darkening observed in diapausing *A. gifuensis*. Several insect cuticular proteins were upregulated at the protein (A0A232F5H6_9HYME) and mRNA (contig_17805, contig_2099, contig_3789, contig_9417) levels in the D group. Transcriptome analysis also identified a chitin synthase (contig_135) that was upregulated during diapause. These modifications thickened and hardened the larval *A. gifuensis* cuticle to improve its resistance to mechanical damage.

### Correlation between transcriptome and proteome

In this study, the correlation between the transcriptome and proteome of the studied species was found to be very low. This phenomenon has been reported in several other studies as well (Tian et al., 2004; Huang et al., 2013). Transcription is an intermediate step in gene expression, and mRNA is the template for protein synthesis. In theory, protein and mRNA expression should be highly consistent. In practice, however, they may not align because of mRNA instability (Waggoner and Liebhaber, 2003), mRNA-ribosome binding (Zong et al., 1999), protein degradation (Beyer et al., 2004), and post-translational protein modification (Futcher et al., 1999). Proteins directly regulate metabolism and physiology. Transcriptome analysis cannot completely reveal protein expression because of post-transcriptional regulation. Proteome studies may also overlook information for degraded or consumed proteins. Transcriptome and proteome analyses are both incomplete, but they could complement each other. For this reason, comprehensive transcriptome and proteome studies have been conducted in diverse research fields in recent years (Vogel and Marcotte, 2012; Kumar et al., 2016).

## Conclusion

In this study, we presented a comprehensive transcriptome and proteome analysis to explore possible molecular processes in *A. gifuensis* diapaus. As a result, we found three important molecular events (carbohydrate accumulation, immune enhancing and cuticle tanning) that may play roles in survival and stress resistance during diapause. Although the conclusions may need further metabology and immunological tests, and numerous other genes and pathways associated with diapause were not analyzed in this study, these findings still provide foundations and directions for the researche of diapause in *Aphidius gifuensis* and, more broadly, even all parasitic wasps.

## Author contributions

H-ZZ, TA, F-XH, and L-SZ designed the research. H-ZZ, TA, and F-XH performed research. Y-YL, M-QW, C-XL, and J-JM provided assistance. H-ZZ, TA, and F-XH analyzed data. H-ZZ wrote the manuscript. Y-YL and L-SZ revised the manuscript.

### Conflict of interest statement

The authors declare that the research was conducted in the absence of any commercial or financial relationships that could be construed as a potential conflict of interest.

## Supplementary material

Figure S1 Original file of **Figure 1A**.

Figure S2 Original file of **Figure 1B**.

Table S1 Expression of the quantified transcript.

Table S2 GO enrichement of differentially expressed genes.

Table S3 GO enrichment of differentially expressed proteins.

Table S4 KEGG enrichment of differentially expressed genes.

Table S5 KEGG enrichment of differentially expressed proteins.

Table S6 UniProt annotation of transcript.

Table S7 Expression of the quantified proteins.

## Abbreviations

D: diapause
ND: non-diapause
PD: post-diapause
DEG: differentially expressed gene
DEP: differentially expressed protein
GO: Gene Ontology
iTRAQ: isobaric tags for relative and absolute quantification
KEGG: Kyoto Encyclopedia of Genes and Genomes.
