# Supplementary figures and images for: Comparative Transcriptome and iTRAQ Proteome Analyses Reveal the Mechanisms of Diapause in Aphidius gifuensis Ashmead (Hymenoptera: Aphidiidae)
Source: Front Physiol. 2018 Nov 30;9:1697. doi: 10.3389/fphys.2018.01697 (PMC6284037; doi:10.3389/fphys.2018.01697)

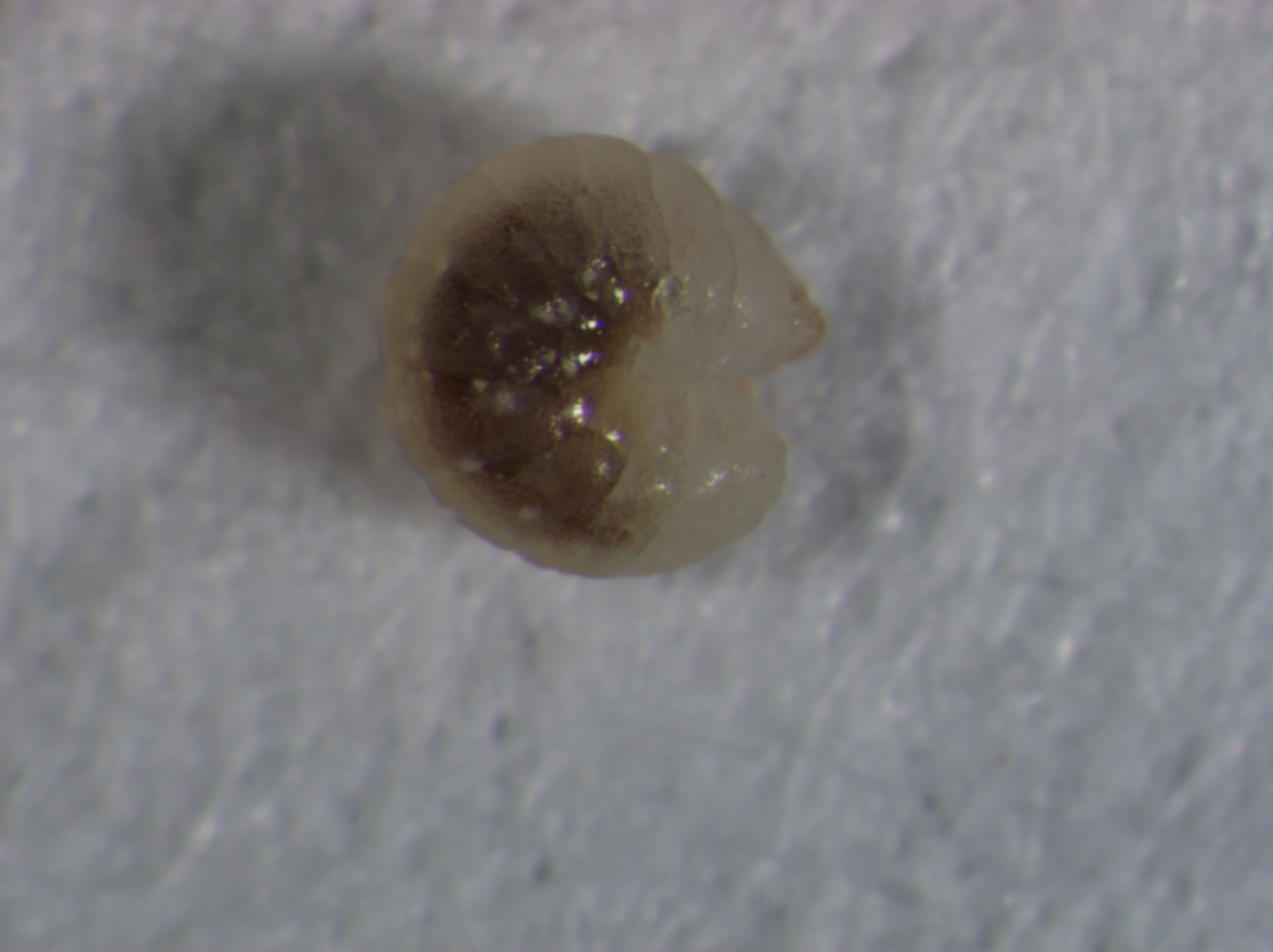

Supplement: Figure S1 — Original file of Figure 1A. [file Image_1.TIF]

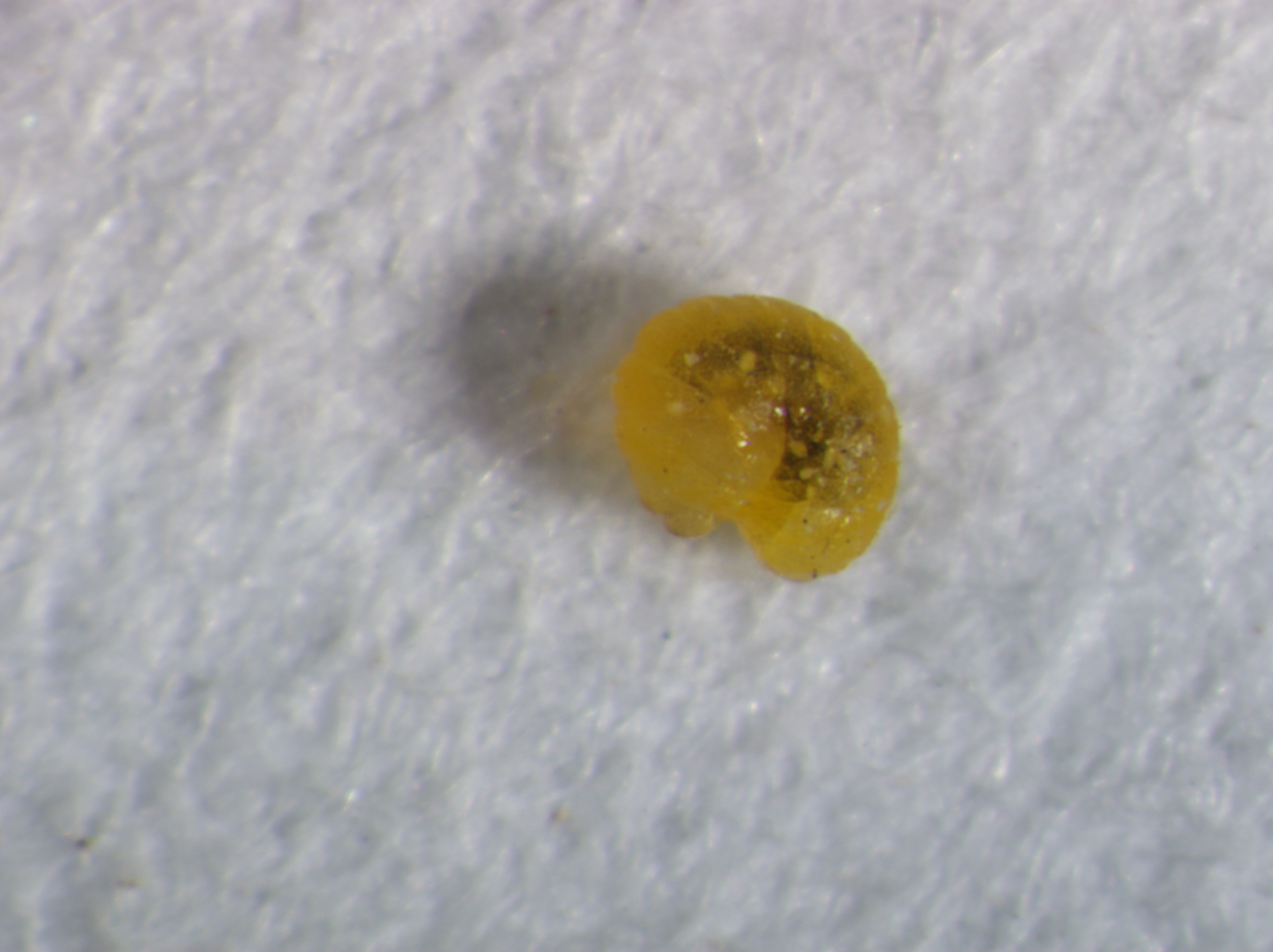

Supplement: Figure S2 — Original file of Figure 1B. [file Image_2.TIF]
